# Patients’ utilisation and perception of the quality of printed health education materials in primary health care: a cross-sectional study

**DOI:** 10.3399/bjgpopen19X101672

**Published:** 2019-11-13

**Authors:** Alhan Haji

**Affiliations:** 1 Consultant Family Medicine and Director of Health Programs and Chronic Disease Department, Administration of Public Health, General Directorate of Health Affairs in Riyadh Region, Ministry of Health, Riyadh, Saudi Arabia

**Keywords:** health education, primary health care, Saudi Arabia, general practice, cross-sectional studies

## Abstract

**Background:**

Printed health education (HE) materials are commonly provided in primary health care (PHC). However, little is known about their use by PHC visitors.

**Aim:**

This study explored patients’ opinions and use of printed HE materials in order to determine an ideal output format for HE.

**Design & setting:**

This was a cross-sectional study, which was conducted in three PHC centres at King Abdulaziz Medical City in Riyadh, Saudi Arabia.

**Method:**

Data were collected through a self-administered questionnaire.

**Results:**

Fifty-five point two per cent of participants obtained printed HE materials from PHC waiting areas. The majority read one or more materials and found it helpful and memorable. Seventy-seven point two per cent applied the written message, 24.0% of participants regularly read HE materials, and more than half spent time reading them in the PHC centre’s waiting area. Around half (51.1%) put the material back in its place after reading it. The preferred format was card with text and graphs. The preferred content was healthy lifestyle advice.

**Conclusion:**

Patients do use printed HE materials in a positive way. More efforts are needed to improve the quality of the materials. Different healthcare providers should contribute more in HE.

## How this fits in

Printed materials are considered one of the main methods for HE. Previous studies have predominantly focused on content, design, and readability of the materials. Having an understanding of how patients use the materials and their preferences will help to improve the delivery of health information, which in turn improves the quality of care at a reduced cost.

## Introduction

People want to participate in their own health care and related health decisions.^1–3^ Healthcare providers are responsible for providing education on health issues and preventive measures. They must ensure the information is sufficient, effective, and positively impacts the quality and cost of care.^1–3^ The key to achieving these goals is HE.

In 1978, the Declaration of Alma Ata put HE as one of the components of PHC. HE was recognised as the most essential component of PHC to reach the goal of *‘h* *ealth for* *a* *ll* *’*.^4^

HE can be provided via different methods, such as verbal one-to-one communication, printed materials, audiovisual materials, and group education such as patient support groups. Each method has its advantages and disadvantages.^2^ Unfortunately, 40%–80% of medical information provided by healthcare practitioners is forgotten immediately.^5^ Moreover, patients remember as little as 20% of the information given to them during a 5-minute consultation, and almost half of the information that is remembered is incorrect.^6,7^ It was found that retention can be increased by up to 50% if there is additional text information after consultations;^8^ therefore, providing written material should be considered.^9^ Patients increasingly expect written material to help them reinforce and recall the verbal information, and enhance their understanding of health issues.^10–12^

In Saudi Arabia, healthcare services are provided through two main sectors: governmental and private; where the Ministry of Health (MOH) is the major governmental provider, with 487 hospitals and 2361 PHC centres.^13^ Other governmental healthcare providers deliver services to a defined population; the National Guard Health Affairs, for example, provides care to its employees, their dependants, and other eligible individuals through five hospitals and 71 PHC centres.^14^ HE departments in the hospitals organise most of the patient HE and promotion efforts.^15^ HE in Saudi Arabia has focused mainly on diabetes management, cardiovascular diseases, the early detection and treatment of cancer, diet and weight management, physical activity, and the prevention or treatment of the diseases in which each hospital specialised.^16^ Consequently, studies regarding HE were also restricted to specific patients groups, such as those with diabetes mellitus, hypertension, or obesity.^17^ The majority of printed HE materials in Saudi Arabia need improvement; therefore, guidelines for designing brochures should be established to improve the quality.^18^

In general, most of the published studies on printed HE materials focused on the content, design, and readability as well as its relationship with the patient's literacy, and the effectiveness of HE on specific patient groups. To the author's knowledge, studies discussing the use of printed materials are few. The literature search did not reveal any published articles that studied the use of printed HE materials by patients in Saudi Arabia.

This study was conducted to: (1) assess the use of printed HE materials by patients in a PHC setting; (2) explore patients’ perceptions and opinions about what good quality HE material should be like, in terms of design, composition, content, and communication style; and (3) determine the preferred modality for HE.

## Method

This cross-sectional study was conducted in three family medicine and PHC centres at King Abdulaziz Medical City of National Guard Health Affairs in Riyadh, the capital of Saudi Arabia. The three centres were:

- A healthcare specialty clinic (HCSC). This is located in the east of Riyadh and serves a population of 350 000.
- King Abdulaziz Housing Clinic (Iskan). This is a clinic in the housing compound for officers and soldiers, and serves a population of 50 000.
- National Guard Comprehensive Specialised Clinic (NGCSC). This is located in the west of Riyadh and serves a population of 180 000.

The study participants were patients and visitors attending the centres mentioned above. They were aged ≥14 years, included both sexes, and were Arabic speakers (only). Exclusion criteria included: illiteracy, mental illness, patients in distress at the time of the interview, and patients who were unable to fill in the questionnaire properly.

The sample size was calculated based on 78% of patients reading the printed items and 80% of patients stating that they found the HE message helpful.^19,20^ The sample size was estimated to be 250 with 0.05 margins of error and 95% confidence intervals (CI) using Epi Info software (version 7). This sample size was distributed among the PHC centres by proportionate method based on the population size of each clinic, with 125 from HCSC, 75 from NGCSC, and 50 from Iskan. Non-random, convenience sampling was used to obtain the sample of the targeted population.

Data were collected through a self-administered questionnaire of 23 items that was created based on the objectives of this study after reviewing the literature. Content validity was ensured through two experienced PHC and research consultants and a health educator reviewing the questionnaire. The translation validity was ensured by using a forward–backward translation method, whereby the questionnaire was first built in English then translated to Arabic by a professional translator who was fluent in both Arabic and English. Another professional translator translated the Arabic version back to English. Then both versions were compared to ensure the accuracy of the translation process. The tool was pre-tested in a pilot study on 10% of the total sample size (25 patients) before collecting the data to ensure the clarity of the questions. Pilot study questionnaires were not included in the final sample.

The questionnaire was divided into four sections. The first section included the demographic characteristics of the study population such as age, sex, marital status, educational level, and health status. Health status was defined as being diagnosed with chronic diseases; for example, diabetes mellitus, hypertension, dyslipidaemia, or asthma. The second section had close-ended (best response and ‘yes or no’) questions about the use of the education materials in the PHC centres, with specification of the numbers of read materials, as well as the usability of and the attitudes towards the read information. The third section had questions exploring the opinion of the participants about what constitutes good quality material from the perspective of design, composition, content, and communication style. And the last section was about the preferred method for HE, other than the printed materials. The questionnaire was distributed to participants after obtaining verbal consent in the waiting area of each centre by a well-trained, Arabic-speaking nurse who explained the questionnaires and collected them once completed.

Data were entered and statistically analysed using Statistical Package of Social Science (SPSS, version 25). Different variables were measured and analysed. The level of statistical significance was set at 95% (**P**<0.05). The tests used for analysis were χ^2^ and student *t*-test.

## Results

The study included 250 participants with no dropout. Table 1 summarises their demographic characteristics. Females represented 60.4% of the sample. The majority were married (70.4%) and half (50.8%) were highly educated (that is, university degree or higher). The majority of the participants (77.2%) were aged <40 years; the mean age was 32 (standard deviation ±10.4) years, with participants ranging in age from 14–68 years. Just over one-quarter of the participants (25.6%) had chronic diseases and 38.0% were on medication for either chronic or acute illnesses. More than half of the participants (55.2%) usually got the printed HE materials from the PHC centres’ waiting areas, while only 18.8% received them from the physicians (Table 2).

**Table 1.** Demographic characteristics of the participants (*n* = 250)

**Characteristics**	***n* (%) **
**Sex**
Male	99 (39.6)
Female	151 (60.4)
**Age, years**
15–25	66 (26.4)
26–40	127 (50.8)
41–60	40 (16.0)
>60	3 (1.2)
Not mentioned	14 (5.6)
**Marital status**
Married	176 (70.4)
Not married	74 (29.6)
**Education level**
Not formally educated	3 (1.2)
School education	120 (48.0)
Higher education	127 (50.8)
**Has chronic disease**
Yes	64 (25.6)
No	186 (74.4)
**On medication**
Yes	95 (38.0)
No	155 (62.0)

**Table 2.** Participants‘ primary source of printed health education materials (*n* = 205)

**Source**	***n* (** **%)**
Physician	47 (18.8)
PHC centre waiting area	138 (55.2)
Nurse	5 (2.0)
Health educator	11 (4.4)
Health education campaign	24 (9.6)
Other	25 (10.0)

PHC = primary health care.

**Figure 1.**
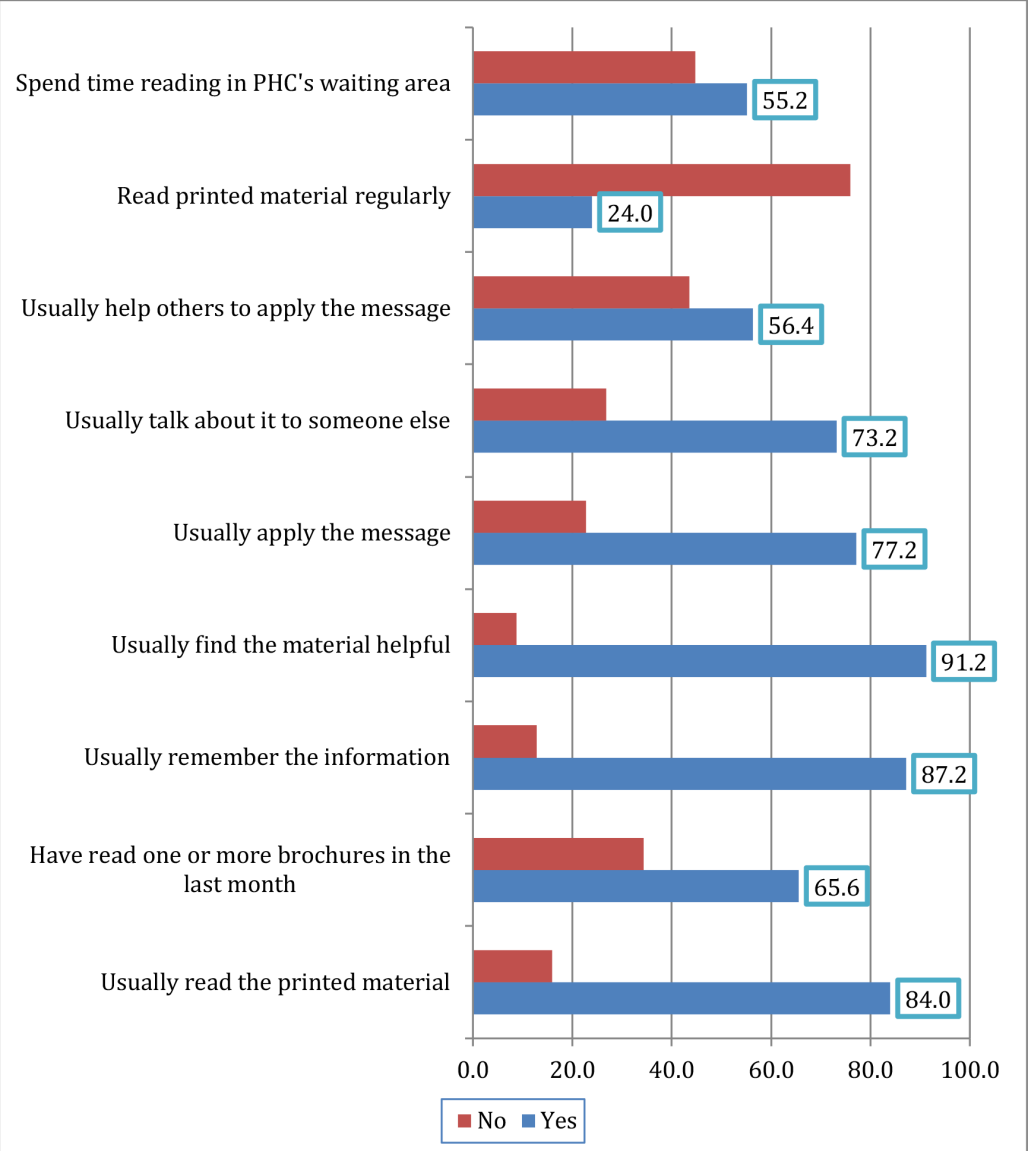
Participants' use of printed health education materials in percentage

**Figure 2.**
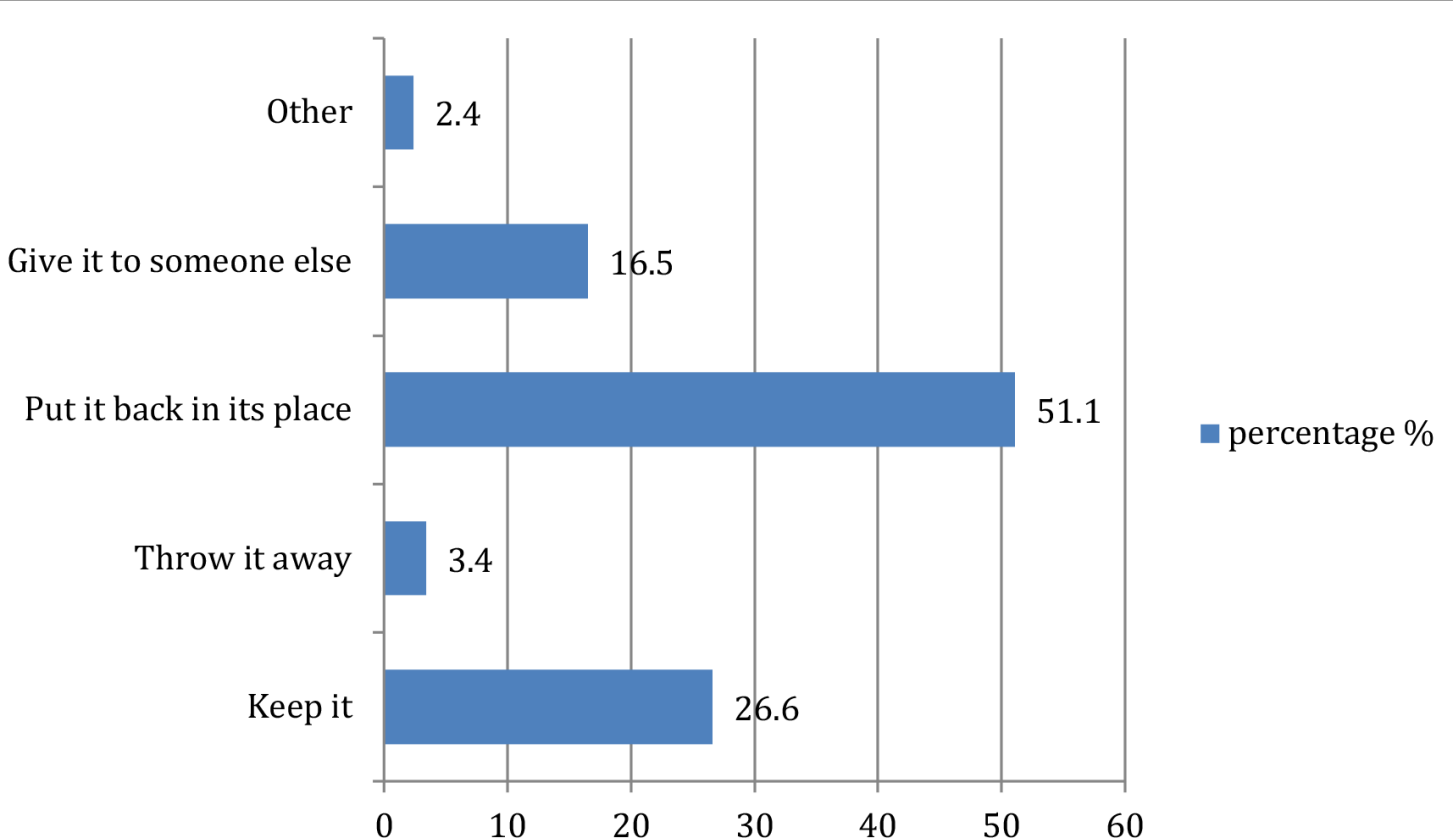
What participants usually do after reading the printed materials

Figure 1 shows how participants used the printed HE materials in PHC settings. Just over half of the participants (51.1%) put the printed materials back in their place after reading them (Figure 2).

How materials were used did not differ significantly in relation to the sex, age, or health status of participants. More than half of participants who stated that they read printed materials were highly educated (χ^2^ = 7.2, *P* = 0.027). It was noticed that the materials were more beneficial for higher educated participants (χ^2^ = 7.05, *P* = 0.029). Participants who talked to someone else about what they have read and those who helped others to apply the message were more likely to be university graduates (χ^2^ = 6.6, *P* = 0.03 and χ^2^ = 7.5, *P* = 0.02, respectively). The majority of participants who used printed materials were married, while 35.6% of unmarried participants stated that they do not usually read the materials (χ^2^ = 30.3, *P* = 0.001). Half of the participants who used the printed materials had chronic illnesses (χ^2^ = 8.4, *P* = 0.014). Nearly 20% of participants who stated that they usually spend time reading materials in the waiting area of PHC clinics had chronic illnesses (χ^2^ = 5.04, *P* = 0.025).

Table 3 explores the participants’ perception of and preferred design for printed HE materials. They preferred them to be formatted as a card (30.8%) or trifold brochure (28.8%). Graphs were the most chosen add-on to text (46.0%). Further analysis indicated that most of the participants who chose card format were female (χ^2^ = 10.7, *P* = 0.03), married (χ^2^ = 10.7, *P* = 0.029), and had school education level (χ^2^ = 18.8, *P* = 0.01). Participants who chose graphs were mainly females (χ^2^ = 11.4, *P* = 0.02), and university graduates (χ^2^ = 25.7, *P*<0.001). Healthy lifestyle and primary prevention was the preferred content or topic (38.8%), while the preferred communication style was in the form of advice (46.0%), with no significant differences according to sex, age, marital status, education level, or health status. Nearly all (93.9%) of the participants, the majority of whom were married, thought that printed HE materials are an effective way to raise health awareness (χ^2^ = 4.3, *P* = 0.037).

**Table 3.** Participants’ preferences for the content and design of printed health education material (*n* = 205)

**Aspect**	***n* (** **%)**
**Format**	
Trifold brochure	72 (28.8)
Plain sheet	38 (15.2)
Card	77 (30.8)
Handbook	54 (21.6)
Other	9 (3.6)
**Add-on**	
Photos	80 (32.0)
Caricatures	13 (5.2)
Tables	23 (9.2)
Graphs	115 (46)
Other	19 (7.6)
**Content**	
Healthy lifestyle & primary prevention	97 (38.8)
Screening & secondary prevention	73 (29.2)
General information about disease	76 (30.4)
Other	4 (1.6)
**Communication style**	
Medical (scientific)	58 (23.2)
Cautionary	45 (18.0)
Emotional	31 (12.4)
Advisory	115 (46.0)
Other	1 (0.4)

**Figure 3.**
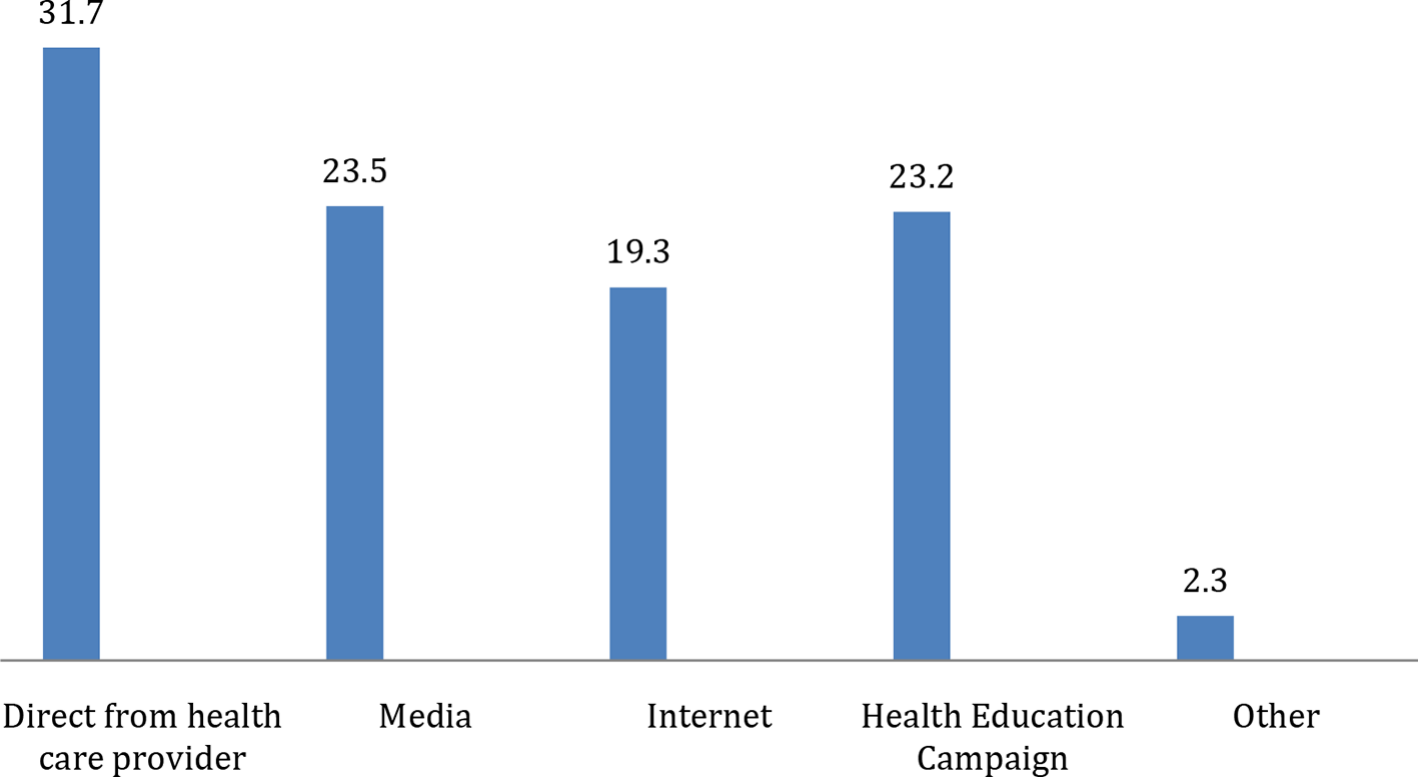
Participants' preferred method of health education other than printed materials in percentage.

Regarding other modalities of HE provision, 31.7% of the participants preferred to be educated directly from a healthcare provider; that is, physician, nurse, or health educator (Figure 3).

## Discussion

### Summary

Printed HE materials are considered one of the most effective ways to raise health awareness, but patients still believe that a direct conversation with a healthcare provider should take place.

### Strengths and limitations

This study was conducted to explore how patients use printed HE materials in PHC centres at King Abdulaziz Medical City, Riyadh, Saudi Arabia. To the author's knowledge, this is the first effort to assess the use of printed HE materials and patients’ perceptions on material quality in a primary care setting locally.

The limitations of this study include the non-random sampling technique, though it was felt that this was the most effective way to overcome the illiteracy and low educational level issue in the population of the National Guard. The use of a self-administered questionnaire might result in a recall bias. Limitations also include social desirability bias because the study was conducted in PHC centres, and the participants might have been inclined to give responses that indicated that they were a health-conscious person, keen to seek health-related information. The study took place in only three PHC centres at one hospital in Riyadh; as a result, it cannot be generalised to cover other hospitals or areas around the kingdom.

### Comparison with existing literature

Results of the current study showed that patients prefer the printed material to be prepared as a card or trifold brochure, which is similar to another study, which indicated that Arab patients prefer trifold brochures with photos.^20^

The participants wanted more materials that provide healthy lifestyle and primary prevention tips. This result is similar to what was found in Al-Khashan *et al,* where 43% of the participants stated that they need education about healthy lifestyle, with significant difference between the needs of men and women on HE related to primary prevention and unhealthy practices (for example, smoking).^21^

Waiting areas at the PHC centres should be utilised, with audiovisual materials in additional to the printed materials. This fits with Maskell *et al*'s recommendation of utilising available technologies to widen access to information.^22^

### Implications for research and practice

The results indicated that waiting areas are considered important sources of printed HE materials in PHC settings, where most of the patients spend a considerable time waiting for their appointments. Meanwhile the role of health educator was less impactful as a source of HE materials, as only 4.4% reported that they had received them from a health educator.

Based on the results, printed HE materials are well used by the majority of participants. However, less than one-quarter of the participants regularly read printed materials. This finding raises questions about the content and quality of the materials meeting all the patients’ needs and preferences. The finding that 51.1% put the printed material back in its place demonstrates the benefit of having reusable materials.

The role of HE campaigns should not be ignored, as around 10% of participants got the materials when attending campaigns and almost one-quarter preferred campaigns as a method for HE.

In conclusion, this study has provided a detailed exploration on how patients use the printed HE materials. The patients’ preferences were for HE cards or trifold brochures with graphs that gave advice on how to live a healthy lifestyle. Quality of printed HE materials should be improved to match the patients’ needs and preferences, and the role of healthcare providers in distributing HE materials should be enhanced. Use of new technology should also be considered. However, the issue of illiteracy should be taken in consideration and more studies should be done to evaluate this issue.
